# Improving Usefulness of Cognitive Decline Population Measures in Predicting Future Dementia Burden

**DOI:** 10.1093/geroni/igab046.692

**Published:** 2021-12-17

**Authors:** Benjamin Olivari, Christopher Taylor, Lisa McGuire

**Affiliations:** 1 Centers for Disease Control and Prevention (CDC), Atlanta, Georgia, United States; 2 CDC, Atlanta, Georgia, United States

## Abstract

Alzheimer’s disease and related dementias begin with mild early symptoms of memory loss, progressing to more severe cognitive and functional impairment. Reports of worsening memory and subjective cognitive decline (SCD) are often the earliest possible signs of dementia onset. The trajectory of certain types of dementia may require early detection of worsening memory in the disease progression for successful interventions. However, the predictive value of subjective measures of cognitive decline is limited; the majority of those who report subjective symptoms do not progress to diagnosed cognitive impairment or dementia. These two realities create a significant challenge in confronting the growing dementia crisis. Population-level data can be beneficial in tracking trends in SCD. Data from the Behavioral Risk Factor Surveillance System (BRFSS) core questions related to chronic diseases and from the SCD optional module from survey years 2015-2019 were aggregated across the participating 50 states, D.C., and Puerto Rico for this analysis. Among 181,097 U.S. respondents aged ≥45 years, 11.3% (95% CI=10.9-11.6) reported SCD; among 20,424 with SCD symptoms, 39.4% (37.6-40.6) reported functional difficulties associated with SCD symptoms and 33.9% (32.4-35.5) needed assistance with day-to-day activities resulting from symptoms. Studies suggest persons experiencing SCD symptoms and associated functional difficulties are at increased risk for dementia compared with those with SCD without functional difficulties. Combining responses about SCD with associated functional difficulties, anxiety, and other measures might help to better inform the future burden of more severe cognitive impairment than SCD status alone.

